# Embryonal rhabdomyosarcoma of the biliary tree in a paediatric patient – A rare cause of obstructive jaundice

**DOI:** 10.4102/sajr.v23i1.1662

**Published:** 2019-01-10

**Authors:** Denny Mathew, Heliodora de Lima, Nasreen Mahomed

**Affiliations:** 1 Department of Radiology, University of the Witwatersrand, South Africa; 2 Helen Joseph Hospital, Johannesburg, South Africa; 3 Rahima Moosa Mother and Child Hospital, Johannesburg, South Africa; 4 Department of Radiology, Rahima Moosa Mother and Child Hospital, Johannesburg, South Africa; 5 South African Society of Paediatric Imaging, Cresta, South Africa

## Abstract

Rhabdomyosarcoma (RMS) is the most common soft-tissue sarcoma in the paediatric age group, ranking fourth in frequency after central nervous system tumours, neuroblastomas and nephroblastomas. Embryonal RMS of the biliary tree is considered a rare entity, with the most common clinical presentation being that of obstructive jaundice. We present the case of a 4-year-old boy who presented with hepatomegaly and obstructive jaundice. Biochemically, there was evidence of elevated ductal enzymes with conjugated hyperbilirubinaemia. The magnetic resonance imaging (MRI) features were consistent with a biliary RMS with the differential diagnosis of a choledochal cyst initially included based on the computed tomography images. The diagnosis of embryonal biliary RMS was later confirmed on histology. This case illustrates the importance of considering malignant aetiologies in paediatric cases of obstructive jaundice, as this entity is infrequently described in the literature and may mimic the appearance of a choledochal cyst. The demonstration of enhancement of intraductal material within the biliary tree on MRI and the presence of arterial waveforms within the intraductal mass on ultrasound assists in the differentiation between biliary RMS and a choledochal cyst.

## Introduction

Rhabdomyosarcoma (RMS) is a malignant tumour of skeletal cell morphology, with the most common sites in the paediatric population being the head and neck, genitourinary system and extremities.^1,2,3^ Embryonal biliary RMS is considered a rare entity with only about 50 cases being described in the literature, the largest being a series involving 25 patients over a period of 25 years.^2^ Given the low incidence of biliary RMS, the diagnosis is challenging, making the imaging evaluation of particular importance in defining the site of origin and tumour extent.^3^ In addition, as a result of the sheltered location of these masses, they are discovered very late, as in our patient, making it difficult to determine the precise organ of origin, and further complicating the diagnosis and potential management.^1^ This case report aims to highlight this diagnosis and justify the inclusion of biliary RMS in the differential diagnoses of a child presenting with obstructive jaundice.^3,4^

## Case report

A 4-year-old boy presented with a 3-month history of yellow discolouration of his eyes, dark urine and pale stools as well as a 2-month progressive history of abdominal distension. On physical examination, he was pale, had scleral icterus and the abdomen was distended with a large palpable liver. On the day of his admission, serology revealed elevated liver ductal enzymes with conjugated hyperbilirubinaemia, an elevated international normalised ratio (INR) and iron deficiency anaemia.

**FIGURE 1:**
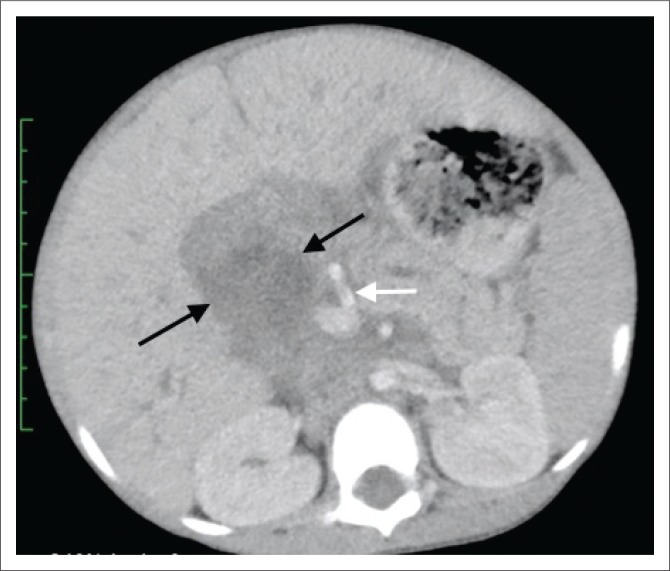
Axial post contrast computed tomography image at the level of the origin of the superior mesenteric artery (white arrow) demonstrating a fusiform-shaped mass (black arrows) with heterogeneous contrast uptake, parallel to the expected course of the common bile duct. Associated findings of prominent intrahepatic bile ducts and a dilated pancreatic duct.

**FIGURE 2:**
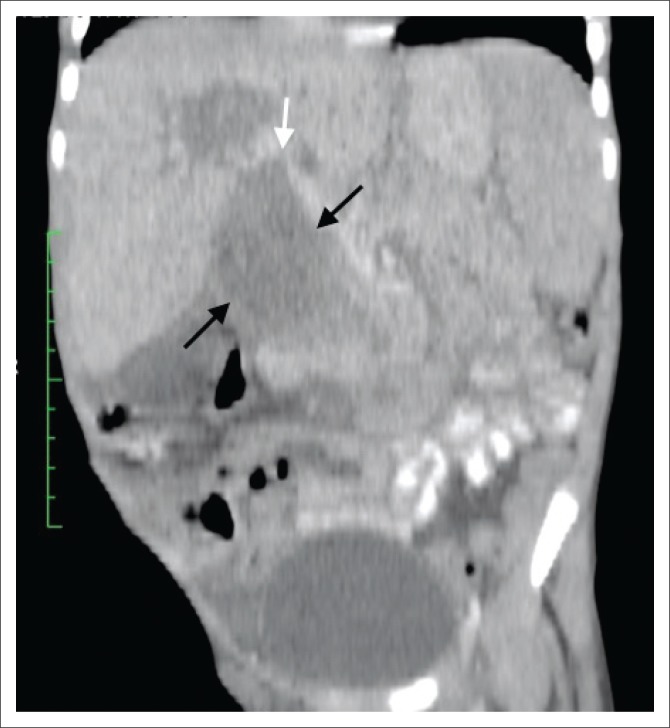
Coronal post contrast computed tomography image demonstrating a heterogeneous mass within the common bile duct (black arrows) with the superior aspect not separable from the adjacent porta hepatis or proximal central bile ducts (white arrow).

The abdominal ultrasound (US) demonstrated a heterogeneous periportal mass with internal flow on colour Doppler and associated dilatation of the common bile duct (CBD), cystic duct, gall bladder and intrahepatic bile ducts. A computed tomography (CT) scan of the abdomen and pelvis with an intravenous and oral contrast agent showed a large (60 mm × 45 mm × 89 mm), fusiform-shaped mass with heterogeneous contrast uptake, parallel to the expected course of the CBD (Figure 1), with the superior aspect not separable from the adjacent porta hepatis or proximal central bile ducts (Figure 2). The portal vein was partially attenuated with no evidence of tumour thrombosis. The medial border of this mass was separable from the pancreas, with the lateral border deviating and partially compressing the D1 and proximal D2 segment of the duodenum with no features of bowel obstruction. Inferiorly, the mass extended just below the inferior pole of the right kidney. The aorta was slightly displaced to the left but not attenuated and the inferior vena cava (IVC) partially attenuated with no tumour thrombus. There was secondary diffuse intrahepatic bile duct dilatation and marked dilatation of the gallbladder and cystic duct. The pancreatic duct was also dilated throughout its course. Ascites was present and there was no evidence of metastases to the liver, lung or visualised bone, but associated enhancing para-aortic and mesenteric nodes were noted. The prostate gland, scrotal sac and contents were normal.

**FIGURE 3:**
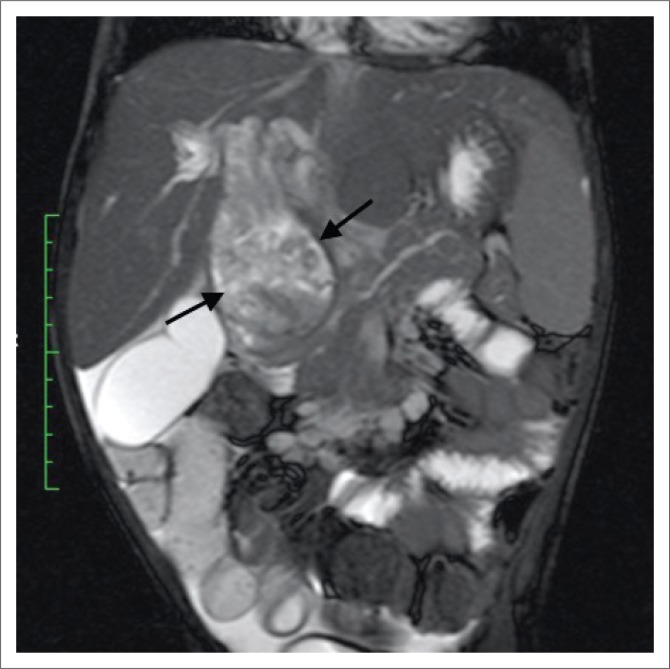
Coronal T2-weighted magnetic resonance imaging illustrating a fusiform-shaped mass arising within the common bile duct (black arrows) extending superiorly to the level of the porta hepatis. This mass is largely hyperintense on T2-weighted imaging with patchy regions of hypointensity, likely secondary to coagulative necrosis.

**FIGURE 4:**
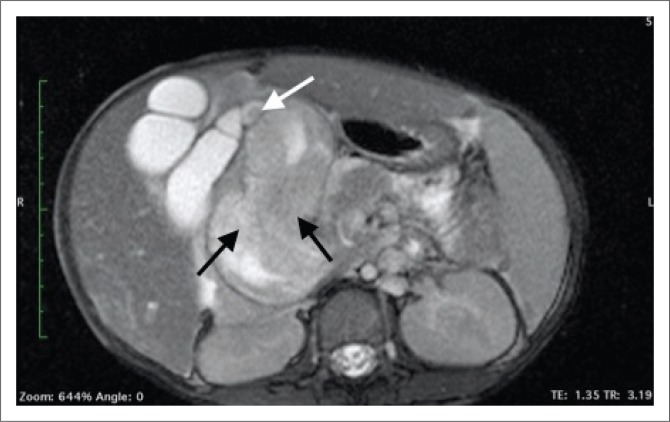
Axial T2-weighted magnetic resonance imaging demonstrating asymmetric mural thickening of the common bile duct (black arrows) with dilation of the gallbladder and a small calculus (white arrow) within the dilated cystic duct.

**FIGURE 5:**
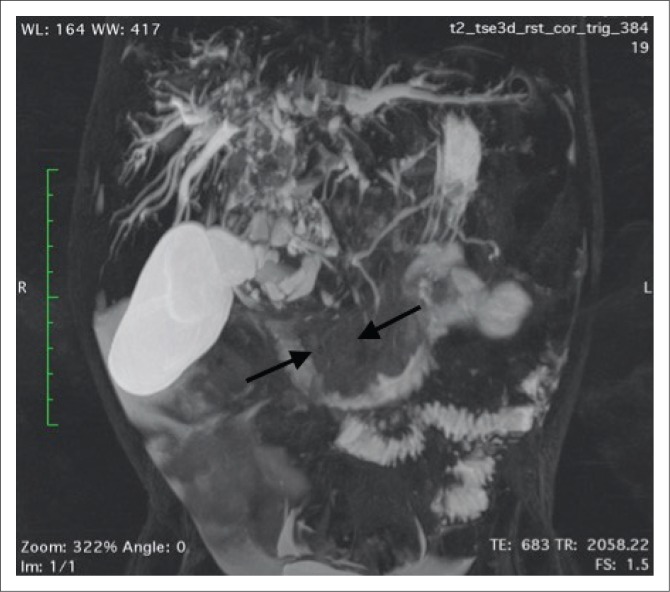
Coronal magnetic resonance cholangiopancreatography shows distension of the gallbladder with dilated intrahepatic bile ducts, cystic duct and pancreatic duct. The common bile duct is dilated with a large filling defect (black arrows) and no normal calibre common bile duct is visualised distally.

Magnetic resonance imaging (MRI) of the abdomen and magnetic resonance cholangiopancreatography (MRCP) with contrast were of added value in better delineating the origin and extent of the mass already suspected of being bile duct in origin on CT. The lesion filled the dilated CBD (Figure 3) and demonstrated asymmetric mural thickening of the CBD (Figure 4). There was associated dilation of the gallbladder and a small calculus was noted within the dilated cystic duct (Figure 4). The contrast-enhanced MRI demonstrated heterogeneous enhancement within the solid components of this mass. Magnetic resonance cholangiopancreatography illustrated a dilated CBD with a large filling defect as well the previously noted findings of dilated intrahepatic bile ducts and pancreatic duct (Figure 5).

The MRI features were consistent with a biliary RMS with the differential diagnosis of a choledochal cyst initially included based on the CT images. The patient was taken to theatre where the ascites was drained and the peritoneal cavity inspected. A solid heterogeneous mass was found within the dilated CBD. In addition, there was a periportal lymph node mass as well as peritoneal wall deposits. Multiple tissue biopsies were sent off for histopathological evaluation.

Microscopy confirmed the presence of a high-grade malignant tumour consisting of primitive mesenchymal cells of varying phases of myogenesis with a variable content of rhabdomyoblasts. The immunohistochemical features were in keeping with embryonal RMS with positive stains of Periodic Acid-Schiff (PAS), desmin, myogenin and myoD1. There were representative sections that showed that the tumour infiltrated into the fibrovascular connective tissue as well as evidence of angiolymphatic infiltration. Perinodal soft-tissue extension was noted; however, no biliary mucosa was present for assessment.

The immunophenotypic features were in keeping with embroyonal RMS with the probability of bile tract origin based on imaging and intraoperative findings. The patient then received post-operative chemotherapy and was initiated on cycle 1 of the VICE protocol which included vincristine, isosfamide, carboplatin and etoposide. Unfortunately, a few days after initiating treatment, the patient became very ill in the ward. His abdomen became very tense and distended which caused splinting of the diaphragm. He subsequently developed respiratory distress and demised.

## Discussion

Rhabdomyosarcoma is the most common soft-tissue sarcoma in the paediatric age group and represents 5% – 10% of all malignant solid tumours in childhood.^1^ Rhabdomyosarcoma ranks fourth in frequency among childhood tumours after central nervous system tumours, neuroblastomas and nephroblastomas.^1^

Embryonal RMS was first described by Wilks and Moxon in 1875 on the basis of the typical location and gross description of the tumour.^4^ In the Intergroup Rhabdomyosarcoma Studies I–IV between 1972 and 1998, of all the cases of RMS, only 0.5% of cases involved the biliary tree.^3^

The International Classification of Rhabdomyosarcoma (ICR) provides a prognostically relevant classification that includes histological subtypes as being of superior prognosis (botyroid and spindle cell RMS), intermediate prognosis (typical embryonal RMS) and poor prognosis (alveolar RMS).^5^ In the paediatric population, the two main histological subtypes of RMS are embryonal and alveolar, with embryonal RMS accounting for nearly 70% of all cases and usually affecting children less than 8 years of age.^1,6^ Histopathologically, RMS is characterised by myoblastic differentiation and expression of skeletal muscle markers, such as desmin, myogenin and/or myoD1.^6^

The median age of presentation of embryonal RMS is 3 years, with a slight male predominance.^7^ The most common clinical presentation is that of obstructive jaundice, which is seen as a presenting symptom in 60% − 80% cases, and may be accompanied by hepatomegaly, abdominal distension and acholic stools.^7,8^ This is frequently also accompanied by elevated liver enzymes and conjugated bilirubin.^3^ Less commonly, pain, nausea, vomiting and fever may also be noticed.^2^ Unlike RMS at other sites, RMS of biliary pathology usually suggests embryonic origin, and in children, there are no neoplasms that arise from the bile duct other than RMS.^7^

Obstructive jaundice in the paediatric population beyond the neonatal period may be secondary to choledochal cysts, choledocholithiasis, strictures as a result of chronic cholangitis and rarely neoplasms such as biliary RMS.^3^

Abdominal US is typically the initial imaging study performed in any patient presenting with obstructive jaundice. In our patient, this demonstrated a soft-tissue mass in the region of the porta hepatis with adjacent mass effect and intrahepatic bile duct dilatation. Computed tomography and MRI/MRCP was then used to better delineate the site of origin, assess the tumour extent and evaluate for metastatic lesions. Magnetic resonance imaging and MRCP confirmed a mass originating within the biliary system that enhanced heterogeneously, followed the signal characteristics of muscle on T1-weighted imaging and was hyperintense on T2WI. Haemorrhage and necrosis may be apparent in larger lesions of biliary RMS and the imaging features of central tumour necrosis may mimic the appearance of a choledochal cyst.^3^

Previous case reports have alluded that biliary RMS is often misdiagnosed as a choledochal cyst on imaging.^3^ Demonstration of enhancement of intraductal material within the biliary tree on MRI and the presence of arterial waveforms within the intraductal mass on US assists in the differentiation between biliary RMS and choledochal cyst filled with sludge.^3^ The presence of regional nodal disease or distant metastases on imaging at the time of diagnosis is of added value in considering a neoplasm as the primary cause of the obstructive jaundice.^3^

The current treatment modalities include a combination of surgical removal, radiation and chemotherapy.^2^ Although gross total excision is not always possible, the prognosis remains relatively good which is probably owing to the favourable histology.^2^ Neoadjuvant chemotherapy followed by resection of the residual tumour has been associated with good outcomes.^3^

## Conclusion

Biliary RMS is a rare entity but should be included in the differential diagnosis of any child presenting with obstructive jaundice.^9^ Imaging plays an important role in the diagnosis and management of this malignancy as well as in differentiating it from a choledochal cyst. The prognosis and long-term survival of biliary RMS has improved with advances in surgery, radiotherapy and chemotherapy.
